# The Gastric Ganglion of *Octopus vulgaris*: Preliminary Characterization of Gene- and Putative Neurochemical-Complexity, and the Effect of *Aggregata octopiana* Digestive Tract Infection on Gene Expression

**DOI:** 10.3389/fphys.2017.01001

**Published:** 2017-12-15

**Authors:** Elena Baldascino, Giulia Di Cristina, Perla Tedesco, Carl Hobbs, Tanya J. Shaw, Giovanna Ponte, Paul L. R. Andrews

**Affiliations:** ^1^Department of Biology and Evolution of Marine Organisms, Stazione Zoologica Anton Dohrn, Napoli, Italy; ^2^Wolfson Centre for Age-Related Diseases, King's College London, London, United Kingdom; ^3^Centre for Inflammation Biology and Cancer Immunology, King's College London, London, United Kingdom; ^4^Association for Cephalopod Research - CephRes, Napoli, Italy

**Keywords:** digestive system, *Octopus vulgaris*, gastric ganglion, parasite, *Aggregata octopiana*, gene expression

## Abstract

The gastric ganglion is the largest visceral ganglion in cephalopods. It is connected to the brain and is implicated in regulation of digestive tract functions. Here we have investigated the neurochemical complexity (through *in silico* gene expression analysis and immunohistochemistry) of the gastric ganglion in *Octopus vulgaris* and tested whether the expression of a selected number of genes was influenced by the magnitude of digestive tract parasitic infection by *Aggregata octopiana*. Novel evidence was obtained for putative peptide and non-peptide neurotransmitters in the gastric ganglion: cephalotocin, corticotrophin releasing factor, FMRFamide, gamma amino butyric acid, 5-hydroxytryptamine, molluscan insulin-related peptide 3, peptide PRQFV-amide, and tachykinin–related peptide. Receptors for cholecystokinin_A_ and cholecystokinin_B_, and orexin_2_ were also identified in this context for the first time. We report evidence for acetylcholine, dopamine, noradrenaline, octopamine, small cardioactive peptide related peptide, and receptors for cephalotocin and octopressin, confirming previous publications. The effects of *Aggregata* observed here extend those previously described by showing effects on the gastric ganglion; in animals with a higher level of infection, genes implicated in inflammation (NFκB, fascin, serpinB10 and the toll-like 3 receptor) increased their relative expression, but TNF-α gene expression was lower as was expression of other genes implicated in oxidative stress (i.e., superoxide dismutase, peroxiredoxin 6, and glutathione peroxidase). Elevated *Aggregata* levels in the octopuses corresponded to an increase in the expression of the cholecystokinin_A_ receptor and the small cardioactive peptide-related peptide. In contrast, we observed decreased relative expression of cephalotocin, dopamine β-hydroxylase, peptide PRQFV-amide, and tachykinin-related peptide genes. A discussion is provided on (i) potential roles of the various molecules in food intake regulation and digestive tract motility control and (ii) the difference in relative gene expression in the gastric ganglion in octopus with relatively high and low parasitic loads and the similarities to changes in the enteric innervation of mammals with digestive tract parasites. Our results provide additional data to the described neurochemical complexity of *O. vulgaris* gastric ganglion.

## Introduction

The gastric ganglion is a prominent feature of the peripheral nervous system in coleoid cephalopods. Gross morphological descriptions of the ganglion are available for *Sepia officinalis* (Alexandrowicz, 1928), *Idiosepius paradoxus* (Shigeno and Yamamoto, 2002), *Octopus vulgaris* (Young, 1967, 1971), and *Eledone cirrhosa* (Isgrove, 1909); illustration and a brief description during development is available for *Sepioteuthis sepioidea* (Shigeno et al., 2001) and *Euprymna scolopes* (Kerbl et al., 2013). In contrast to the single gastric ganglion in coleoid cephalopods, in *Nautilus* a pair of small ganglia distributing nerves to the viscera emerge from the visceral nerves (Owen, 1832).

The gastric ganglion (see original description for *O. vulgaris* in: Chéron, 1866; Bogoraze and Cazal, 1946) innervates most of the digestive tract, i.e., the crop, stomach, intestine, and caecum. It also connects with the central nervous system via the sympathetic nerves, the visceral nerves through rectal and intestinal nerves and through the abdominal nerves (Young, 1967). The complex structure of the gastric ganglion and its relationships support the view that it functions both independently and integrating information originating from, for example, the crop and intestine (Young, 1967), thus appearing to act not only as a simple relay but also as an integrative center (Andrews and Tansey, 1983). The intricate connectivity and complexity of the ganglion is further revealed by intense tubulinergic immunoreactivity of the neuropil (e.g., Shigeno and Yamamoto, 2002).

The well-defined innervation of the cephalopod digestive tract and the fact that it often hosts parasites (review in: Hochberg, 1983; Castellanos-Martínez and Gestal, 2013) raise the possibility that the presence of parasites may induce physiological responses (e.g., Gestal et al., 2002b) in the innervation, as occurs in mammals (see below).

In mammals, digestive tract pathogens (i.e., bacteria, viruses or parasites) can induce a range of responses including local inflammation, sensitization of visceral afferent nerves (peripheral terminal, cell body and central nervous system levels) and modulation of enteric nervous system (ENS) functionality (for review see: Halliez and Buret, 2015; Guarino et al., 2016; Obata and Pachnis, 2016). Examples are provided by the bacteria *Campylobacter jejuni* (Goehler et al., 2005), *Clostridium difficile* (Wadhwa et al., 2016) and *Salmonella typhimurium* (Gabanyi et al., 2016), rotavirus (Lundgren et al., 2000; Istrate et al., 2014) and the parasites *Giardia duodenalis, Nippostrongylus brasiliensis, Trypanosoma cruzi* and *Trichinella spiralis* (for review see Halliez and Buret, 2015). Mucosal damage of the digestive tract, such as occurs with an ulcer, can also produce sensory neuron sensitization (Bielefeldt et al., 2002), as can intestinal inflammation (Stewart et al., 2003).

With parasitic infections, changes observed in the gut innervation in rodents (mice or rats) include increased levels of the tachykinin substance P (e.g., *Trichinella spiralis*, Swain et al., 1992; *Nippostrongylus brasiliensis*, Masson et al., 1996), reduced acetylcholine release (e.g., *Trichinella spiralis*, Collins et al., 1989), and acute and chronic selective damage to the ENS (*Trypanosoma cruzi*, Campos et al., 2016). The sensitization of visceral afferent neurons and damage to the enteric innervation produced by pathogens contribute to post-infectious syndromes in humans (e.g., post-infectious gastroparesis in children, Naftali et al., 2007; post-infectious irritable bowel syndrome in adults, Schwille-Kiuntke et al., 2015; Wadhwa et al., 2016). Thus, the innervation of the mammalian digestive tract is affected both acutely and chronically by a range of pathogens including parasites.

In *O. vulgaris* the presence of parasites (review in: Hochberg, 1983; Castellanos-Martínez and Gestal, 2013) may induce responses either locally or systemically. The parasite most frequently found in octopus digestive tract is *Aggregata octopiana* (Estévez et al., 1996; Castellanos-Martínez and Gestal, 2013), a microscopic Coccidian, spore-forming, single-celled obligate intracellular parasite. It is one of the various species of *Aggregata* belonging to apicomplexan Protozoa (Apicomplexa: Aggregatidae). *A. octopiana* may reach incidences higher than 90% in some populations of *O. vulgaris* (e.g., West Mediterranean, Mayo-Hernández et al., 2013). In the digestive tract of octopus, *A. octopiana* is found in both non-cuticularized (caecum and intestine), and cuticularized (esophagus and crop) structures, in the digestive gland and other nearby organs (Gestal et al., 2002a,b). In infected animals, cysts are visible with the naked eye as small white patches embedded in the muscular wall of the digestive tract (Mayo-Hernández et al., 2013). Therefore, they are in close proximity to the enteric neurons located in the serosa and between the circular and longitudinal muscle layers of the digestive tract in cephalopods (Alexandrowicz, 1928; Graziadei, 1960).

Histological and ultrastructural lesions associated with *A. octopiana* infection include hypertrophy with nuclear displacement of the host cell, inflammation, phagocytosis, ulceration and final damage of the organ architecture (Gestal et al., 2002a). *Aggregata* appears to be particularly abundant in the caecum and intestine in the octopus, and has been proposed to be responsible for a “malabsorption syndrome” (Gestal et al., 2002b). *A. octopiana* has also been suggested to induce a systemic immune response (Castellanos-Martínez et al., 2014a; Gestal and Castellanos-Martínez, 2015).

## Aim of this study

The effects of the lesions associated with *A. octopiana* infection in *O. vulgaris* have been explored at different levels, but the impact on neural structures has never been investigated. Here we ask whether *Aggegata* affects one component of the digestive tract innervation, namely the gastric ganglion. Our hypothesis is based on the assumption that the gastric ganglion is connected, through both sensory and motor neurons, to all regions of the digestive tract, which can be affected by *Aggregata*, and therefore the damage and local inflammation induced by the parasite will cause functional modification reflected in gene expression. The neurons of the gastric ganglion could also be affected by substances (e.g., TNFα) released as components of the systemic immune response to *Aggregata* (Castellanos-Martínez et al., 2014a,b).

To explore our working hypothesis and to expand current knowledge of the neural complexity of this ganglion, we: (i) surveyed the neurochemical diversity of the gastric ganglion in *O. vulgaris* by a combination of approaches including *in silico* molecular characterization and immunohistochemistry with a particular focus on ligands and receptors likely to be involved in neurotransmission; (ii) used real time RT-qPCR to compare the expression of selected target genes in the gastric ganglion from octopuses with relatively “high” and “low” *Aggregata* parasite loads, to provide insights into the potential impact of infection on the control of the digestive tract.

## Materials and methods

### Animals and tissue sampling

*Octopus vulgaris* Cuvier, 1797 (Mollusca, Cephalopoda) of both sexes (males, *N* = 10 − body weight: mean ± SEM = 185 ± 22 g; females, *N* = 12 − body weight: mean ± SEM = 272 ± 19 g) were obtained from local fishermen (Bay of Naples, Italy). Twelve octopuses were used for immunohistochemistry, and an additional 10 utilized for analysis of the potential effects of *Aggregata* infection on the gastric ganglion (see below). All animals originated from a larger sampling study measuring the incidence of octopus' parasite load (*A. octopiana*) in the Mediterranean.

Killing animals solely for tissue removal does not require authorization from the National Competent Authority under Directive 2010/63/EU and its transposition into national legislation. Samples were taken from local fishermen, by applying humane killing following principles detailed in Annex IV of Directive 2010/63/EU as described in Fiorito et al. (2015). In brief, octopuses were immersed in freshly made 3.5% magnesium chloride hexahydrate (Sigma Aldrich, CAS Number: 7791-18-6) dissolved in sea water. After 30 min immersion, the animals were unresponsive to handling and a noxious mechanical stimulus, and ventilation completely stopped; killing was completed by destruction of the brain.

### Tissue removal

**Figure 1:**
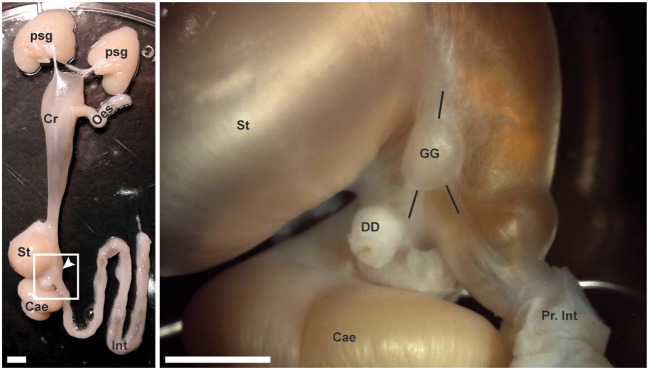
The gastric ganglion of *Octopus vulgaris*. **(Left panel)** A dissected digestive tract from an octopus. From top (anterior) to bottom (posterior): Posterior salivary glands (Psg), crop (Cr), esophagus (Oes), stomach (St), caecum (Cae), and intestine (Int). Within the square (see magnification in the **Right panel**), the arrow points to the gastric ganglion. **(Right panel)** In higher magnification, the same structures are identified together with the gastric ganglion (GG) and surrounding nerve bundles (see black lines to highlight their relative positions) running toward the stomach and crop (up), and toward the caecum and digestive duct (bottom left) or toward the intestine (bottom right). The proximal part of the intestine (Pr. Int) and the cut end of the digestive duct (DD) are also evident. Scale bars: 500 μm.

All dissections were carried out on an ice bed. The digestive tract was approached via incision of the dorsal mantle and of the capsule covering the digestive gland. The gastric ganglion was identified on the right-hand side at the junction of the crop, stomach, caecum and intestine (Figure 1). The ganglion was removed by transection of nerve trunks projecting to adjacent structures and cutting the connective tissue capsule adherent to the serosa of the digestive tract. The ganglion was placed initially in either fixative (for immunohistochemistry) or RNA Later Stabilization Solution (RNALater, Thermo Fisher Scientific; for gene expression studies) as indicated below.

In addition, the entire digestive tract was removed by transection of the esophagus as it exited the brain and the rectum at the level of the anal sphincter. Blunt dissection freed the gastric ganglion from adherent structures.

For all animals, the digestive tract was inspected for the presence of white cysts indicative of *A. octopiana* infection and rapidly frozen on dry ice and then stored at −20°C for subsequent quantification of the magnitude of *A. octopiana* infection (see below).

### Immunohistochemistry and staining

Excised gastric ganglia from animals with no visible *Aggregata* infection were immersed in fixative appropriate for each of the employed antisera and/or treated for the specific purpose of the required staining (Table 1). In most cases for paraffin and cryostat sections ganglia were placed in 4% paraformaldehyde (PFA) in seawater (4°C for 2 h). After fixation, the tissue was treated as briefly summarized below.

- Transferred to 70% ethanol (in distilled H_2_O) until processing and paraffin-embedding (~2 weeks) according to standard protocols (i.e., slow dehydration in increasing concentrations of ethanol, clearing in xylene overnight, three changes through liquid paraffin, and embedding). The embedded ganglia were then sectioned at 5–7 μm and dried overnight at 60°C.
- For cryosectioning after several washes in PBS, ganglia were cryoprotected in sucrose 30% and embedded in OCT compound (Sakura Tissue-Tek OCT Compound; Gentaur, San Jose, CA). The embedded ganglia were then sectioned at 20 μm.
- To allow detection of noradrenaline and octopamine, we followed the protocol developed by Ponte and Fiorito (2015) for octopus. In brief, the tissue was fixed (6.25% glutaraldehyde, 75% picric acid, 5% glacial acetic acid, and 1% sodium metabisulfite in distilled water; 3 h), washed in sodium metabisulfite solution (SBM) and dehydrated in an ascending ethanol series until permeabilization (mixture of ethanol/methyl salicylate, 5 min) and then rehydrated in a descending ethanol series (in Tris-HCl-SMB) and embedded in agarose. The embedded ganglia were then sectioned at 50 μm (Vibratome VT1200S, Leica Microsystems, Germany) and collected as free-floating sections. No antigen retrieval was required (for details see Ponte and Fiorito, 2015).

**Table 1:** List of antibodies utilized in this study and their source.

**Antigen**			**Methods**	**Supplier**	**Catalog number**	**References**
**Full name**	**Abbreviation**	**Ab type**				
Acetylated alpha-tubulin	Ac-Tub	M	PFA – Paraf	Sigma	T 6793	Shigeno and Yamamoto, 2002; Wollesen et al., 2009
Neurofilament 200	NF	M	PFA – Paraf	Sigma	N 5389	Imperadore et al., 2017
Neuronal nuclear antigen (RNA binding protein fox-1 homolog 1)	NeuN	P-Cy3 Conjugate	PFA – Paraf^a^	Millipore	ABN78C3	Present study
Corticotropin Releasing Factor	CRF	P	PFA – Cryo	Peninsula Labs	T-4037	Suzuki et al., 2003
FMRFamide (FMRF-BTg)	FMRFamide	P	PFA – Cryo	Immunostar	20091	Di Cristo et al., 2003; Wollesen et al., 2012
Tyrosine Hydroxylase	TH	P	PFA – Cryo	Millipore	AB152	Baratte and Bonnaud, 2009; Ponte, 2012
Noradrenaline (NA-G-BSA)	NA	P	(Ponte and Fiorito, 2015; see also Methods section)	GemacBio	AP006	Ponte, 2012; Ponte and Fiorito, 2015
Octopamine (OA-G-BSA)	OA	P	(Ponte and Fiorito, 2015; see also Methods section)	GemacBio	AP007	Ponte, 2012; Ponte and Fiorito, 2015
γ-Aminobutyric acid (GABA-BSA)	GABA	M	PFA – Paraf	Sigma	A0310	Cornwell et al., 1993; Ponte et al., 2010
5-Hydroxytryptamine (5-HT-BSA)	5-HT	P	PFA – Paraf	Sigma	S5545	Ponte et al., 2010; Wollesen et al., 2010, 2012
Substance P (TAC1-BSA)	TAC1	M	PFA – Cryo	Novusbio	NB100-65219	Springer et al., 2004
Common type of Choline Acetyltransferase	cChAT	P	(Casini et al., 2012)^b^	Dr JP Bellier (gift)		Casini et al., 2012

*Name and abbreviation and type of antibody (monoclonal: M, polyclonal: P) utilized are included together with supplier and catalog number. Cited references refer to previous studies where the same antibody (or with identical epitope) has been utilized for nervous tissue in cephalopods*.

a *Antibody applied after antigen retrieval as described by Herculano-Houzel and Lent (2005): 1 h (70°C) in 0.2 M boric acid solution (pH 9). The sections were then washed in PBS and incubated with a primary antibody against NeuN (rabbit polyclonal; Cy3 conjugate)*.

b *To detect cChAT-ir we followed recommendations provided by Casini et al. (2012)*.

For fluorescence immunohistochemistry (IHC) on paraffin tissue, sections were deparaffinized, rehydrated and subjected to heat-mediated antigen retrieval in Sodium Citrate buffer (pH 6.0). Sections were blocked in 10% goat serum (2h at RT) and then incubated at 4°C with primary antibody overnight. After washing (PBS, 3 × 5 min) tissues were incubated with AlexaFluor-conjugated secondary antibodies as appropriate for 2 h at 37°C in the dark. For the final 15 min, DAPI was added for detection of DNA/nuclei. Tissue was again washed in PBS (3 × 5 min), and then mounted with fluorescent mounting medium (Fluoromount™ Sigma Aldrich).

For NeuN detection we applied antigen retrieval to paraffin sections as described by Herculano-Houzel and Lent (2005), i.e., boric acid solution (see Table 1 for details) and then incubated with a primary antibody against NeuN (rabbit polyclonal; Cy3 conjugate).

To detect cChAT immunoreactivity we followed the standard protocol utilized for octopus (Casini et al., 2012).

The omission of each primary antibody was used as a negative control for each immunostaining procedure.

Classic histological staining was utilized to examine the general organization of the octopus' gastric ganglion. We applied Hematoxylin and Eosin (H&E) on deparaffinized/rehydrated tissues incubated in Mayer's hematoxylin (Sigma) for 5 min, then washed in running tap water (5 min), followed by incubation with Eosin Y (Sigma) solution (1 min), and then rapidly dehydrated, cleared in xylene, and mounted (Mayer, 1893; e.g., Shigeno et al., 2001). Serial sections (50 μm) were also collected on chrome-alum-gelatin-coated slides, and stained using with Picro-Ponceau with hematoxylin following Kier (1992).

Classical histological sections were examined using bright-field microscopy (Zeiss Axiocam 2, with Zen-Blue software) and photographed with a Zeiss105 color camera. For IHC we utilized a Leica DMI6000 B inverted microscope and a Leica TCS SP8 X confocal microscope (Leica Microsystems, Germany). Tile Z-stacks were performed using a 0.2 μm step size. Images were processed using LAS X software (Leica Microsystems, Germany). IHC figures have been assembled following guidelines for color blindness provided by Wong (2011).

### Assessment of *Aggregata octopiana* infection

The entire digestive tract (esophagus to rectum) of *O. vulgaris* (*N* = 10) was removed immediately *post mortem*, weighed and after vigorous washing in homogenization buffer processed according to Gestal and co-workers (Gestal et al., 1999; see also Gestal et al., 2007; Tedesco et al., 2017). In brief, after homogenization, sporocysts of *A. octopiana* were counted by a Neubauer chamber and their number expressed as sporocysts per gram of tissue. Samples considered in this study were taken from a larger sampling study aimed to describe possible differential parasite loads in different octopuses. We selected samples to assure a significant difference in parasite load between animals (see section Results).

### Transcriptome analysis and *in silico* characterization of the gastric ganglion

A preliminary characterization of the complexity of the gastric ganglion of *O. vulgaris* was conducted utilizing recent transcriptome data derived from Drs. R. Sanges' and G. Fiorito's Research Groups at the Stazione Zoologica Anton Dohrn (Napoli, Italy). We utilized a dataset based on two separate RNA-seq studies (Petrosino, 2015; Zarrella et al., unpublished data) carried out on *O. vulgaris* central nervous system (i.e., optic lobes, supra-esophageal and sub-esophageal masses), distal extremities of arm (including muscular and nervous tissues), stellate and gastric ganglia (for technical details see: Musacchia et al., 2015; Petrosino, 2015). The resulting transcriptome (Drs. R. Sanges and G. Fiorito Labs: Stazione Zoologica Anton Dohrn; see also Petrosino, 2015) identified more than a hundred thousand transcripts from different neural structures, significantly extending previously available transcriptome data for this species (Zhang et al., 2012).

We applied a biased strategy, based on Gene Ontology (http://www.geneontology.org/), to these datasets and mined for gene-transcripts potentially involved and/or implicated in the response to infection, inflammation, immune/stress responses.

### Analysis of gene expression

#### RNA extraction and cDNA synthesis

Gastric ganglia were dissected from animals and stored in RNA Later Stabilization solution for 24 h (+4°C) and transferred to −80°C until further processing. Total RNA was extracted using SV Total RNA Isolation System (Promega, Z3100) according to manufacturer instructions. Quality and quantity of extracted RNA was assessed through UV absorption measurements (Nanodrop ND-1000 UV-Vis spectrophotometer, Nanodrop Technologies). Absence of DNA contamination was verified through PCR (ubiquitin primers) followed by gel electrophoresis. For cDNA synthesis, 500 ng of total RNA from each sample were processed with iScript-cDNA Synthesis Kit (Bio-Rad, 1708891).

#### Primer design: efficiency and specificity

Primers were designed by Primer3Plus software (www.bioinformatics.nl/primer3plus) using target sequences deduced from *O. vulgaris* transcriptome (Petrosino, 2015). Primer parameters were set to 20 nucleotides in length, product size 100–200 base pairs and melting point 58–60°C. Primers were also analyzed with the Multiple Primer Analyzer^1^ to estimate presence of, and possibly avoid, primer-dimers.

The efficiency of each pair of primers (Table 2) was calculated according to standard methods curves, and following Sirakov et al. (2009). Briefly, five serial dilutions (1:5, 1:10, 1:20, 1:40, 1:80) of a standard sample were made to determine the efficiency of reactions conducted with each pair of primers. Standard curves were generated for each sample/gene combination using the *Ct* value vs. the logarithm of each dilution factor (Pfaffl et al., 2002; Radonić et al., 2004).

**Table 2:** *O. vulgaris* genes (*n* = 33) identified and utilized in this study.

**Protein name**	**Gene name**	**AN**	**Primer sequence 5′-3′**		**AS**	**E**	**GO annotation**		
							**Process**	**Function**	**Component**
Glutathione synthetase	Gss	P48637	F2	CAGGATTGGGAAACTCGGAAAA	218	2.00	0006520	0000166	0005829
		(MF467271)	R2	ACAGCTTCCTCTCCTTCAGG					
Nuclear factor NF-kappa-B p100 subunit	Nfkb2	Q9WTK5	F	ACTGCAGCAAGGGTATTTGC	96	1.86	0000122	0000977	0005634
		(MF467265)	R	TACGCTTTGTCACGTGCATC					
Adenosine receptor A2a	ADORA2A	P29274					0001963	0001609	0005882
		(MF467261)							
Tool like receptor 3	Tlr3	Q0PV50	F	CGCTCTTTGGATCTTTCTGG	134	2.00	0001819	0003723	0005768
		(MF467268)	R	AATGTGTTGCTGCTGACACG					
Toll-like receptor 13	Tlr13	Q6R5N8					0002376	0003723	0005737
		(MF467260)							
Allograft inflammatory factor 1-like	Aif1l	Q9EQX4	F	TGTTGTTGTGATCCCCCTCT	139	1.74	0008150	0003779	0005737
		(MF414196)	R	TTGCCTGCAAGTGTTACTCG					
Protein singed	Sn	Q24524	F	AGGGCAAATATGCACTGGTC	117	1.95	0007015	0003779	0005737
		(MF467264)	R	AGATTTGACCCGACTTGAGC					
Neurabin-1	PPP1R9A	Q9ULJ8					0007015	0003779	0005737
		(MF467258)							
Dopamine beta-hydroxylase	Dbh	Q64237	F	ATCCGGAAGGAGTGAATGTG	185	2	0001816	0003824	0005615
		(MF414198)	R	GAAGCGCTGGAAGTTTTGTC					
Peroxiredoxin 6	Prdx6	Q5ZJF4	F	CCTTCTGTGCGTGAAGATGA	104	2	0006629	0003824	0005615
		(MF467270)	R	TTTGGACAAGCTGTCATTCG					
Endothelin-converting enzyme 1	ECE1	P97739					0006508	0004222	0005886
Glutathione S-transferase omega-2	Gsto2	Q6AXV9	F2	CCGCATGAGATAGTGAACATCA	173	2	0006749	0004364	0005737
		(MF467266)	R2	TCAGTGGGAGTTAATGGTGGA					
Glutathione peroxidase	GPX	U5XKR1	F	ACGTGCAGATGAACAAGCTG	198	2	0001659	0004601	0005737
		(KF233998)	R	GAGCGTTTTCTCCGTTGACT					
Superoxide dismutase Cu-Zn	Sod1	P80566	F	CCACAGGCAAGTCTTTTCCC	192	2	0006801	0004784	0005623
		(MF467269)	R	CGACCTTGGCAATGTTGAGT					
Serpin B10	Serpinb10	P48595	F2	GAGGTCAATGAAGAAGGTACGG	91	1.93	0010466	0004867	0005615
		(MF467263)	R2	ATTTCAGTGGTGGCATCCTC					
Gastrin/cholecystokinin type B receptor	Cckbr	P46627	F	ATCGAGTGGCACGTTTATCC	101	1.96	0007165	0004871	0005886
			R	GAAGTAACACCGCGAAGCTC					
Cholecystokinin receptor type A	Cckar	Q63931 (KY994648)	F	CTAGACCCATGGCCGTTCTA	141	1.88	0007165	0004871	0005886
			R	CTGCTGGCCTACACCTCTTC					
Octopressin receptor	OPR	Q5W9T5	F	CGCCTTGGATCGTTACATCT	124	1.74	0007165	0004871	0005886
		(AB116233)	R	CCAGTTGTGGCAATGAACAC					
Orexin receptor type 2	Hcrtr2	O43614	F	CAAGCAAGGACGAAGGTAGG	105	1.96	0007165	0004871	0005886
		(MF467262)	R	GTCATCAAAAACGGCCAGAT					
Corticotropin-releasing factor receptor 2	Crhr2	Q60748					0007165	0004871	0005783
		(MF467256)							
Cephalotocin receptor 1	CTR1	Q7YW31					0007165	0004871	0005886
		(AB158493)							
Lipopolysaccharide-induced TNF-alpha factor	Litaf	Q8QGW7	F	CTCGTTGCCCCAAATGTAAC	105	2	0001816	0005125	0005737
		(MF467267)	R	AACTGAGGAGGAGGCAAATG					
Molluscan insulin-related peptide 3	Mirp	P80090	F	TGAGAGGGGATGAGGAGATG	190	2	0008284^*^	0005158^*^	0005576^*^
			R	AACTGGAGTGGGATCGTTTG					
Cephalotocin	CT	Q76DN0	F	ACCCCTTTCACAGAACATGC	142	1.92	0001975^*^	0005179^*^	0005576^*^
		(AB162925)	R	TGTATGTCTTGCGGACCAAA					
Fatty acid-binding protein homolog 9	lbp-9	Q965W1	F	AACGACCATGTCCCTAAACG	122	1.92	0006810	0005215	
		(MF414199)	R	CGGGCTGCTCTATACCACTC					
High-affinity choline transporter 1	CG7708	Q9VE46 (MF467259)					0003333	0005215	0005887
									
Anoctamin-1	Ano1	Q8BHY3	F	GAACTCATTCGCCAAGAAGC	171	2	0006810	0005227	0005737
		(MF414197)	R	AGGCAGTTTCGTCTGCAGTT					
Sodium- and chloride-dependent glycine transporter 2	SLC6A5	Q9Y345					0006810	0005328	0005826
		(MF414200)							
Rab effector Noc2	Rph3al	Q58D79	F	CCATGAAGGTGGCAAGTTTT	133	1.94	0006886	0005509	
		(MF467257)	R	AACCTTCCTTGTTGCCATTG					
Small cardioactive peptide-related peptide	SCPRP	Q2V2G5	F	AACCCGTATTTGGCGTAGTG	173	2	0007218		0005576
		(AB198190)	R	GTTGCCCTGGTCTGAAAAAG					
Protein PRQFV-amide	PRQFV	Q86MA7	F	CAGTGTTGCGGAGAGATGAA	159	1.89	0007218		0005576
			R	CTGCAGCATGGTCTGACTGT					
Tachykinin-related peptide	Octtkrpre	Q6F6I8	F1	CGTTTAGTTGGGGCTTTTCA	118	1.92	0007218^*^		0005576^*^
		(AB037112)	R1	GGGTTCCCGAGGTAAAAGAG					
Hypothetical protein OCBIM_22024610mg	OCBIM_22024610mg	A0A0L8H1B4					0009607		0016020

Transcripts are given together with valid protein and gene names, and Uniprot accession number (AN); GeneBank accession numbers for O. vulgaris orthologs of these genes are indicated in parenthesis. Whenever available and for nucleotidic sequences that have been validated we provide GenBank accession number (AN, in parenthesis). The table also include sequences of primers (n = 24, Forward and Reverse) used for RT-qPCR experiments, amplicon size (AS) and efficiency (E). Gene ontology (GO) annotation for biological processes, functions and components are included. GO identifiers marked with

* *refer to cases where GO were attributed following the identification of similar proteins known in vertebrates or invertebrates to have analogous functions and when GO does not provide any result based on the valid Protein Name*.

Each amplification reaction was conducted in a volume of 25 μl containing: 2 μl of diluted cDNA template, 2.5 μl of 10 × PCR reaction buffer (Roche), 2.5 μl of dNTP mix (0.2 mM), 1 μl of each primer (25 ρmol/μl), 0.25 μl of Taq DNA polymerase (5U/μl), and sterile H_2_O. The amplification cycles were conducted by Peltier Thermal Cycler PTC-200 (MJ Research). After denaturation at 95°C (2 min) 34 amplification cycles were carried out as follows: denaturation (94°C, 15 s), annealing (60°C, 30 s), extension (72°C, 1 min). Finally, an extension cycle was carried out at 72°C for 7 min to complete all the strands. PCR products were run on 2% agarose gel in TBE buffer 0.5 × (45 mM Tris-borate, 1mM EDTA) and detected expected bands were isolated. DNA was extracted using GenElute Gel Extraction Kit (Sigma-Aldrich, NA1111) and analyzed using an Automated Capillary Electrophoresis Sequencer 3730 DNA Analyzer (Applied Biosystems).

#### Real-time qPCR

In order to analyze expression levels of specific genes of interest, a panel of putative reference genes was first screened to find the most stable genes for these experimental conditions (see the approach utilized for octopus in Sirakov et al., 2009). In our experiments, we utilized: eukaryotic translation initiation factor 4 (*EIF4G1*), LIM and SH3 domain protein (*F42H10.3*), lamin-B1 (*Lmnb1*), cytoplasmic FMR1 (*Sra-1*), ubiquitin-40S ribosomal protein S27a (*RPS27A*), elongation factor 1-alpha (*eef1a*), 40S ribosomal protein S18 (*RPS18*). The gene expression stability of the candidate reference genes for our samples was evaluated with BestKeeper (Pfaffl et al., 2002) and NormFinder (Andersen et al., 2004), following Sirakov et al. (2009). We identified the three most stable reference genes as *Lmnb1*, Sra-1 and *RPS27A*.

For gene expression experiments, samples from 10 octopuses were processed in triplicate. Polymerase chain reactions were carried out in an optical 384-wells plate with Applied Biosystems ViiA7 (Life Technologies) using Fast SYBR Green Master mix (ThermoFisher Scientific) to monitor dsDNA synthesis. Reactions (total volume: 10 μl) contained: 1 μl cDNA, 5 μl SYBR Green Master mix reagent, 4 μl of forward and reverse primers mix (0.7 pmol/μl each). The following thermal profile was used: 95°C for 10 min; 95°C for 15 s, 60°C for 1 min, 40 cycles for amplification; 72°C for 5 min; one cycle for melting curve analysis, from 60° to 95°C to verify the presence of a single product. Specificity of PCR products was checked by melting curve analysis followed by gel electrophoresis and DNA sequencing. PCR data were analyzed using the ViiA™ 7 Software (Life Technologies) to determine cycle threshold (*Ct*) values. Each assay included a no-template control for every primer pair. All sequences have been deposited in GenBank after validation (Table 2).

### Data analysis

Relative expression of the genes of interest, identified by *in silico* analysis of the octopus transcriptome, in the gastric ganglion and other tissues was analyzed through hierarchical clustering and principal component analysis (PCA) using Multi-experiment Viewer (MeV) software (Saeed et al., 2003). Quantitative real-time PCR experiments, carried out to evaluate the response of the octopus gastric ganglion to different levels of *A. octopiana* infection (“high” vs. “low” parasite load), were analyzed through Multivariate Analysis of Variance (MANOVA) following Tsai and Chen (2009). Gene expression changes are expressed as Log_2_ fold-changes following common practice (Friedman et al., 2006; Fundel et al., 2008). For all statistical analyses we used SPSS (rel. 18.0, SPSS Inc. - Chicago, 2009), with the exceptions mentioned above, and following Zar (1999). All tests were two-tailed and the alpha was set at 0.05.

## Results

### *In silico* comparison of gastric ganglion transcripts with other tissues

**Figure 2:**
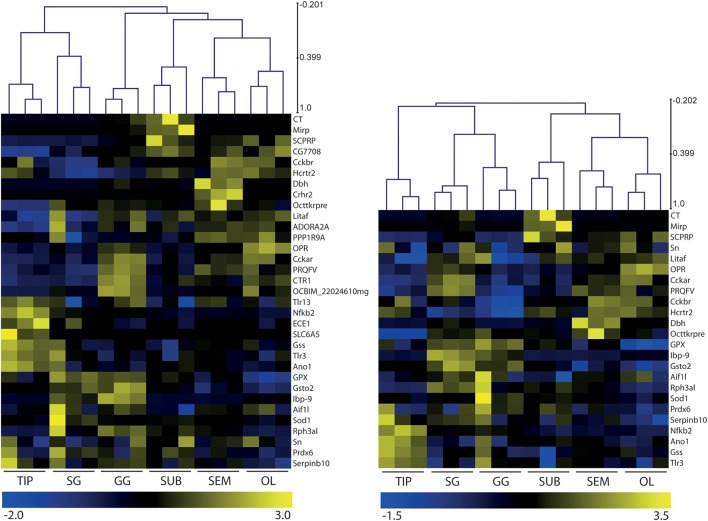
Heatmaps and hierarchical clustering of gene expression levels in octopus brain (OL, SEM, SUB), stellate (SG) and gastric (GG) ganglia, and arm tip (TIP). Thirty-three **(Left panel)** and 24 transcripts **(Right panel)** mined from *Octopus vulgaris* RNAseq data were selected with a focus on neurotransmitters, signaling machinery and inflammation. Data were normalized per gene and subsequently analyzed by hierarchical clustering of the samples (tissues) and transcripts. Differences in the relative expression and clustering revealed a differential pattern between the gastric ganglion and other tissues considered independently from the number of transcripts included in this preliminary *in silico* characterization of octopus gastric ganglion. See Table 2 and text for details.

Figure 2 illustrates the transcriptional profiles of 33 genes expressed in *O. vulgaris* gastric ganglion (Table 2), selected through Gene Ontology (GO) Biological Functions search of the available transcriptome data. In brief, we counted over 65,000 nucleotide transcripts selectively expressed in the octopus' gastric ganglion. Our biased GO searching strategy probed for transcripts considered of particular relevance to the response to infection (e.g., neurotransmitter-synthesizing enzymes, receptors for transmitters and hormones), or implicated in inflammatory and/or immune/stress responses (e.g., superoxide dismutase, toll-like receptors, fatty acid-binding homolog 9); this provided a reduced number of potential candidate genes.

A more comprehensive analysis of the transcriptome fingerprints of *O. vulgaris* gastric ganglion is beyond the scope of this study, and this list should be considered preliminary.

We found that *in silico* relative abundance (counts) of the 33 transcripts in the octopus gastric ganglion varied. These ranged from <1 CPM (endothelin converting enzyme 1) to >1,000 CPM for fatty acid-binding homolog 9 (Lbp-9), the highest count for any transcript considered in any tissue. We also found 16 gene transcripts with counts <10 CPM, and nine in the range 10–100 CPM (allograft inflammatory factor, SCPRPamide, Protein PRQFV-amide, glutathione S-transferase, glutathione peroxidase, superoxide dismutase, peroxiredoxin 6 protein, glutathione synthase-like isoformX3; hypothetical protein OCBIM). Finally, 6 of the 33 genes considered had transcript counts in the range 100–1,000 CPM (dopamine beta-hydroxylase, tachykinin related peptide, Rab effector Noc2, cephalotocin, molluscan insulin-related peptide 3, protein singed). *In silico* relative expression of these 33 transcripts are overviewed in Figure 2.

A subset of 24 of the 33 genes (Table 2) was validated through real time RT-qPCR (see also **Figure 6**). In addition, and to confirm homogeneity between the two sub-sets of *O. vulgaris* gastric ganglion transcripts, we produced heatmaps and performed hierarchical clustering analysis (Figure 2). Differences were identified in the transcriptional fingerprint of *O. vulgaris* gastric ganglion when compared with other tissues, namely the stellate ganglion, arm tip and brain regions (Figure 2). The differences emerged independently from the number of transcripts considered (i.e., either 33 or 24 genes; Figure 2).

**Figure 3:**
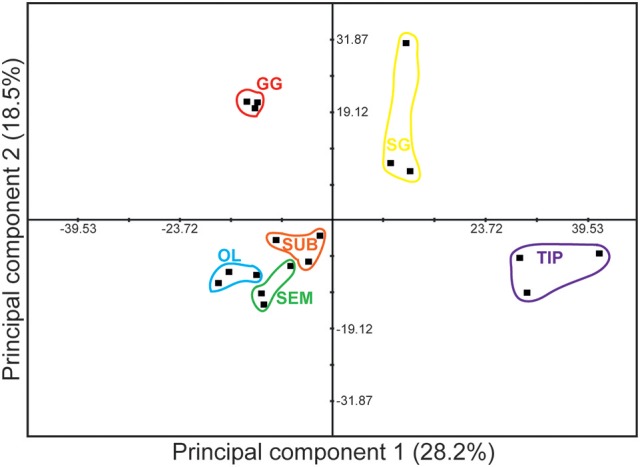
Principal Component Analysis of gene expression data based on transcript levels (*in silico*, see Figure 2) of 33 genes selected for this study (see Table 2 for full list). Data are plotted for tissue taken from three (*N* = 3) different animals. Dissimilarity between peripheral and central nervous system tissues emerged from the analysis with a clear distinction of the octopus gastric ganglion signature from other peripheral structures (i.e., stellate ganglion and arm). See text for details.

Principal Component Analysis (PCA) confirmed that the gastric ganglion profile differs from the other tissues (Figure 3). The two first principal components accounted for less than 50% of the total variance, and the cut-off eigenvalue (set to 1) was achieved only with principal component 5 (for PC analysis of either 33 or 24 transcripts, data not shown). Nevertheless, the gastric ganglion segregated into a different quadrant from the stellate ganglion. Furthermore, the analysis showed that the arm tip and the regions belonging to octopus' central nervous system also segregated in different quadrants (Figure 3), thus confirming that the genes considered here have a specific expression profile, and revealing that the three different brain regions (optic lobes and supra- and sub-esophageal masses) cluster together, but distant from the peripheral tissues considered.

### Extending current knowledge of *O. vulgaris* gastric ganglion structural complexity

**Figure 4:**
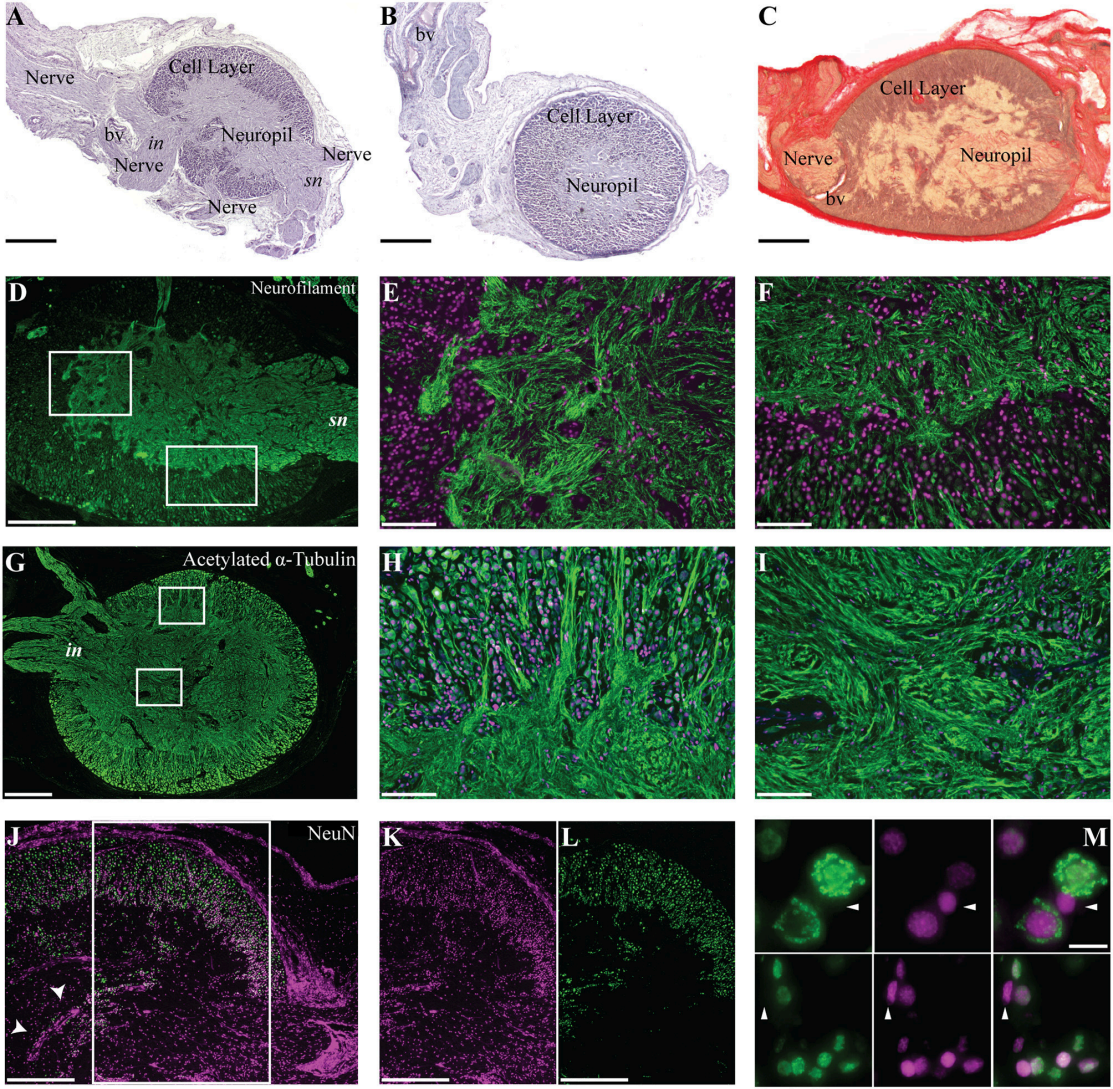
Morphological description of the octopus gastric ganglion. Hematoxylin and eosin **(A,B)** and Picro-Ponceau with hematoxylin **(C)** served to characterize the ganglion, with its cortical layer of neural cells (Cell layer) and internal neuropil. Nerve bundles are highlighted; in **(A)** intestinal (in) and sympathetic (sn) nerves are indicated; staining also revealed blood vessels (bv). Connective tissue and the cell layer surrounding and enveloping the ganglion is clearly identified after Picro-Ponceau with hematoxylin staining **(C)**. **(D–M)** Fluorescent immunohistochemistry for (in green) neurofilament **(D–F)**, acetylated alpha-Tubulin **(G–I)**, and NeuN **(J–M)**, DAPI is utilized as nuclear stain (magenta). Neurofilament marks the inner neuropil **(D)** and the intricate organization of fibers (enlargement in **E**), with some neural processes entering the cellular layer (enlargement in **F**). Acetylated alpha-tubulin **(G–I)** shows the external group (external layer) of cells larger than those distributed internally (internal layer) **(H)** with several processes progressing toward the internal neuropil **(H,I)**. NeuN and nuclear stain (DAPI) merged image **(J)** are provided as separate channels in **(K,L)**, respectively for areas corresponding to the white rectangle. A cell wall dividing the neuropil of the ganglion is clearly visible (**J**, arrowheads). Higher magnification to show the neurons positive for NeuN (green), DAPI (magenta), and merged channels in **(M)**. The cells not positive for NeuN are indicated with arrowheads. Scale bars: 500 μm **(D,G,J,K,L)**; 200 μm **(A–C)**; 100 μm **(E,F,H,I)**; 10 μm **(M)**.

*O. vulgaris* gastric ganglion is an ovoid structure prominently located at the junction of the crop, stomach, caecum and proximal intestine at the point where they share a common lumen (Figure 1). The well-circumscribed nature of the ganglion was particularly evident following histological examination, which confirmed previous data reporting that it is almost entirely encapsulated in connective tissue (Bogoraze and Cazal, 1946; Figures 4A–C). The ganglion itself consists of very densely packed nerve cell bodies distributed in a cortical layer of the oblate spheroid structure, with axons coursing centrally, bundling, and allowing the ganglion to be connected with other structures including the subjacent digestive tract (Figures 4A–C). Following Bogoraze and Cazal (1946), the peripheral layer of cells appears relatively larger than those that are more closely distributed toward the internal neuropil (see also Young, 1971).

Numerous cells also appear in the neuropil. A group of them are clustered together, toward the center of the ganglion, creating a wall apparently separating it into two sections (Figure 4J, arrowheads; see description in Young, 1971). In addition, some cells are dispersed in the neuropil and have been described by Bogoraze and Cazal (1946) as forming a glio-vascular network (Figures 4E,I). In our samples, we found cells with somata diameters ranging from 7 to 30 μm.

In the following section, a further description of the cellular components and fibers constituting the octopus gastric ganglion, together with information derived from attempts to localize modulators and other characteristic markers, is provided through immunohistochemistry (IHC). The results should be considered preliminary. This because the currently available commercial antibodies we utilized are not designed for octopus or cephalopods. However, as shown in Table 1 (consider also exceptions therein) these have been already applied to cephalopod tissues in a series of studies, despite that in some cases standardized ways to biologically validate them have not being applied. Therefore, observations provided below should be considered as an indication of “-like immunoreactivity” (referred hereunder as like-IR or IR). These limitations are considered further in the Discussion.

We utilized NeuN for the first time in octopus. It positively identified the great majority of cells in the gastric ganglion (Figures 4J–M) confirming its general architecture (see above). In addition, there were a few cells negative for the NeuN-antibody (Figure 4M, arrowheads), suggesting also the existence of supporting cells (e.g., glia-like cells) in the gastric ganglion. A complex organization of fibers was revealed through neurofilament-IR; these appeared mostly ordered and compacted into bundles in the part proximal to the sympathetic (Figure 4D, *sn*) nerve (i.e., superior and dorsal bundles, *sensu* Bogoraze and Cazal, 1946). On the other hand, fibers appeared greatly intertwined toward the posterior area of the neuropil (Figure 4D, left rectangle in; enlargement in Figure 4E), before the emergence of the ventral nerve bundles (see also intestinal nerves in Young, 1967). It is in this area that neurofilament-IR revealed fibers forming polygonal, round or ovoid networks resulting from the convergence of numerous fiber bundles (Figures 4D–F).

Using acetylated alpha-tubulin antibody we found positive cells and fibers (Figures 4G–I). The fibers appeared intertwined and arranged in different directions and orientations. Most of them appeared to emerge from larger cells forming networks where cells appeared dispersed into bundles of different size and complexity (Figures 4F,H).

To gain a broader understanding of the molecular complexity and signaling potential of the gastric ganglion, the expression of a range of putative neurotransmitters was also analyzed by IHC.

**Figure 5:**
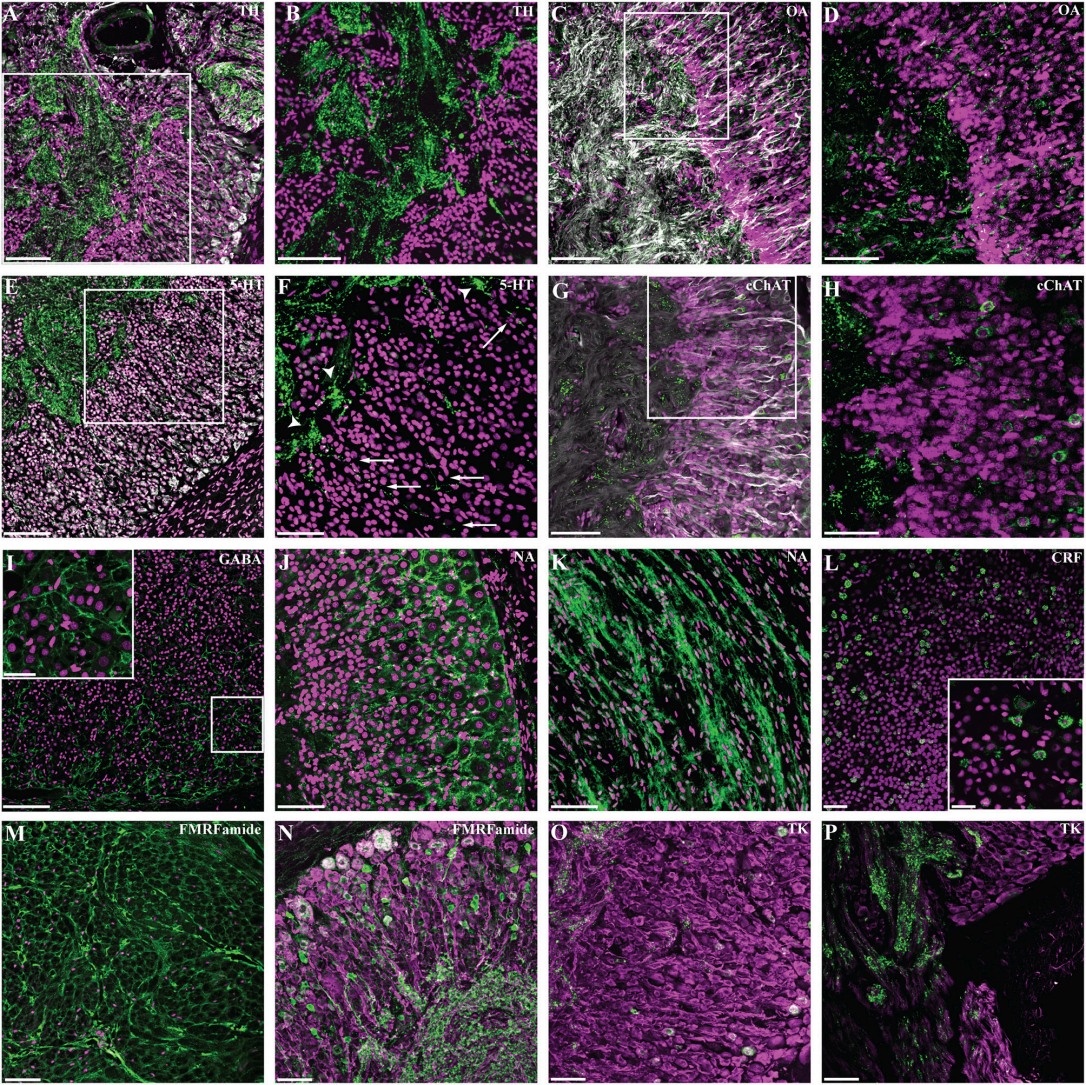
Neurochemical complexity of *Octopus vulgaris* gastric ganglion. Fluorescent immunoreactivity (in green) for: Tyrosine Hydroxylase (TH, **A,B**), Octopamine (OA, **C,D**), 5-hydroxytryptamine (5-HT, **E,F**), common Choline Acetyltransferase (cChAT, **G, H**), GABA **(I)**, Noradrenaline (NA, **J, K**), Corticotrophin releasing factor (CRF, **L**), FMRF-amide **(M,N)**, and Tachykinin (TK, **O**,**P**); DAPI is utilized as nuclear stain (magenta, except in **N–P**). Co-localization with acetylated alpha-tubulin (AcTub) is shown in **(A,E,N,O,P)** with neurofilament (NF) in **(C,G)**. TH-IR fibers as they appear in the internal neuropil of the gastric ganglion **(A)** co-localized with AcTub (gray). The white square area in **(A)** is enlarged in **(B)** to show the strong TH immunoreactivity at the level of neural processes. **(C)** The intricate network of octopamine positive fibers and button-like positivity as seen in the neuropil and cellular layer (colocalized with NF, gray). The high magnification in **(D)** shows details of octopamine-IR fibers inside the cellular layer and their prolongation within the internal neuropil. **(E)** 5-HT-IR and AcTub (gray) revealed fibers in the internal neuropil with several processes progressing toward the cellular layer. The high magnification detail in **(F)** shows some of these processes in the cellular layer (arrows), and some clustered network fibers (arrowheads). **(G)** The common Choline Acetyltransferase immunoreactivity seen in cells and fibers, co-localized with NF (gray). The magnification in **(H)** to highlight cChAT-IR in cells. GABA-IR **(I)** identified an intricate network surrounding the cells of the cortical layer; a magnification is provided in the enlargement. **(J)** Noradrenaline immunoreactivity revealing an intricate reticulum surrounding cells of cellular layer, and at the level of the sympathetic nerve **(K)**. CRF-IR positive cells **(L)** and detail in the higher magnification (square) of the cellular layer. FMRFamide-IR fibers are observed in the intestinal nerve (transverse section, **M**). FMRFamide-IR fibers in the neuropil of the gastric ganglion **(N)** with processes and cells in the cellular layer (co-localized with AcTub, magenta). Tachykinin-like IR in cells and fibers in the neuropil **(O,P)**; co-localization with AcTub (magenta). See text for details. Scale bars: 100 μm (**A,B,D**,**E**,**H**,**I,J**,**K**,**L**); 50 μm (**C**,**G**,**F,M,N,O,P**); 25 μm (higher magnification in **L**,**I**).

We observed numerous nerve fibers positive for tyrosine hydroxylase antibody (Figures 5A,B). These are identified as ordered bundles in the neuropil of the ganglion and toward the anterior pole in areas belonging to the sympathetic nerve bundles. This pattern contrasts with the diffuse and apparently disorganized arrangement observed toward the posterior end, corresponding to the area where neurofilament-like IR revealed intricate round (or ovoid) network of fibers.

A diffuse, but evident and widely distributed IR-signal occurred in sections using octopamine antibody. Octopamine immunoreactivity revealed small “button-like”-vesicles (Figures 5C,D) widely distributed in the cytoplasm and neuronal processes, again suggesting the existence of an intricate network.

5-hydroxytryptamine-like positive nerve fibers (Figures 5E,F) were clearly visible in the neuropil, in some cases forming a clustered network (Figure 5F, arrowheads). Furthermore, we identified several processes originating from larger neurons and progressing toward the neuropil, in some cases surrounding cells belonging to the internal layer of the ganglion (Figure 5F, arrows).

Common type of choline acetyltransferase (cChAT) antibody identified a few sparse, but clustered positive cells (Figures 5G,H), and several positive fibers dispersed in the neuropil.

GABA-like IR revealed an intricate widely distributed reticulum surrounding the great majority (almost all) of cells; this appeared particularly evident in the cells belonging to the more external layers of the cortical zone (Figure 5I). It overlapped with the noradrenaline-like IR we observed (Figure 5J), although appearing less intricate when compared with GABA-like IR. Noradrenaline-IR is also found in fibers of the nerve bundles (Figure 5K).

Corticotrophin releasing factor (CRF-like IR) was identified in a subset of the cells within the cortical layer of the gastric ganglion (Figure 5L) and also in a few CRF-like IR positive nerve fibers.

FMRFamide-like immunoreactivity was widely distributed in the ganglion with numerous positive fibers identified in various parts of the neuropil and in several cells distributed in the surrounding cortical layer (Figures 5M,N). In several areas, strong FMRFamide-like IR was seen in the neuropil in a cluster of fibers forming a beaded appearance (Figure 5M). Furthermore, positive FMRFamide-like fibers have been identified in the nerves that connect the gastric ganglion with other structures.

Various cells of the internal zone of the cortical layer of the gastric ganglion were positive for tachykinin–like immunoreactivity (Figure 5O). We also observed numerous tachykinin-like positive fibers both in the neuropil and nerves (Figure 5P).

### Gene expression in the gastric ganglion as a result of *Aggregata octopiana* infection

**Figure 6:**
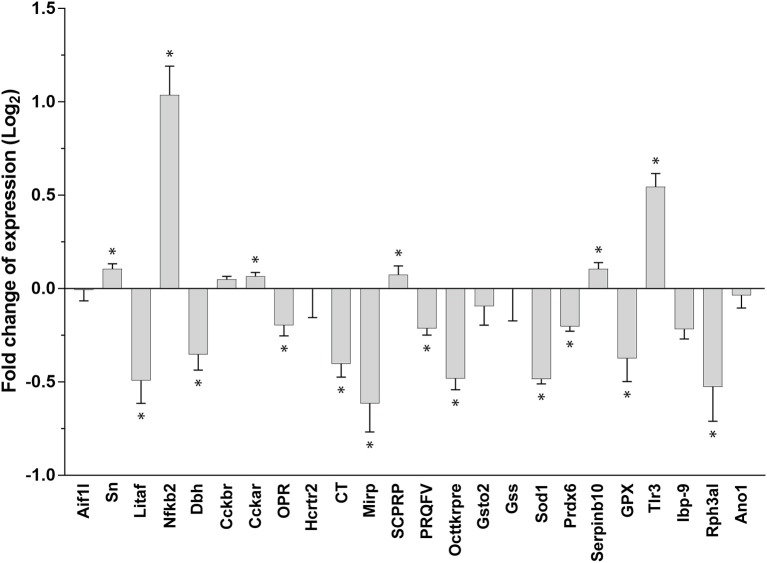
Relative gene expression using real-time RT-qPCR. Fold changes of expression (log_2_, error bar: 95% confidence interval). A differential pattern of up- and downregulation of 24 genes in the octopus gastric ganglion of animals infected by *Aggregata octopiana* (“high” level) emerged when compared (“high”/”low”) with their expression in samples taken from octopus where the parasite load from *Aggregata* was defined as “low.” Asterisks mark significant changes; see text for details and Table 3.

We found diversified expression of the selected 24 genes in the gastric ganglion after RT-qPCR experiments linked to parasite load in the digestive tract samples (Figure 6; see also Table 3). We compared gene expression in gastric ganglia of octopuses with an elevated number of *A. octopiana* sporocysts [“high,” sporocysts/tissue(g): median = 1.37^*^10^6^, 95% CI ± 1.80; *N* = 5] in their digestive tract, with those from *O. vulgaris* with a relatively low parasite load [“low,” sporocysts/tissue(g): median = 0.26^*^10^6^, 95% CI ± 0.21; *N* = 5; parasite incidence, “high” vs. “low”: *Z* = 2.6, N1, N2 = 5, *p* = 0.009 after Mann-Whitney *U* test]. In the latter group, we counted fewer than 6,000 sporocysts per gram of tissue; in “high” group we found the highest value of *Aggregata* sporocysts [sporocysts/tissue(g) = 5.8^*^10^6^]. In three out of ten animals we found a very few (<3/octopus) larval forms of other parasites, i.e., cestodes and nematodes. There is no evidence for effects of these parasites on the functioning of the cephalopod digestive system, to the best of our knowledge (Hochberg, 1990).

We observed a significant change in gene expression in the gastric ganglia in response to infection [high vs. low-−24 genes, MANOVA: *F*_(4, 7)_ = 202.59, *p* < 0.001]. Figure 6 plots gene expression as fold changes observed in octopus gastric ganglia from “high” relative to those sampled from “low” parasite load animals. Seventeen out 24 genes had significant pairwise changes (after Bonferroni *post-hoc* comparison; Figure 6; Table 3). Under these computational circumstances, six genes increased their relative expression (high vs. low: *Sn, Nf*κ*b2, Cckar, SCPRP, Serpinb10, Tlr3*) while decreased gene expression was observed in the others (*n* = 11; high vs. low: *Litaf*, *Dbh, OPR, CT, Mirp, PRQFV*, Tk, *Sod1, Prdx6*, Gpx1, *Rph3al*). Furthermore, genes for Ov-Nuclear factor NF-κB p100 subunit and Ov-Toll like receptor 3 were found with at about one-fold increase (Figure 6). At least a one-fold decrease was observed for Ov-Lipopolysaccharide-induced tumor necrosis factor-alpha factor, Ov-Molluscan insulin-related peptide 3, Ov-Superoxide dismutase [Cu-Zn] (Ov-*Sod1*), and Ov-Rab effector Noc2 (Figure 6).

**Table 3:** Pairwise comparison of gene expression in the gastric ganglion between “high” vs. “low” *Aggregata octopiana* infected octopuses.

**Protein**	**Gene**	***P* (after Bonferroni)**
Allograft inflammatory factor 1-like	Aif1l	0.729
Protein singed	Sn	**<0.001**
Lipopolysaccharide-induced TNF-alpha factor	*Litaf*	**<0.001**
Nuclear factor NF-kappa-B p100 subunit	*Nfκb2*	**<0.001**
Dopamine beta-hydroxylase	*Dbh*	**0.001**
Gastrin/cholecystokinin type B receptor	*Cckbr*	0.051
Cholecystokinin receptor type A	*Cckar*	**0.01**
Octopressin receptor	*OPR*	**<0.001**
Orexin receptor type 2	*Hcrtr2*	0.318
Cephalotocin	*CT*	**<0.001**
Molluscan insulin-related peptide 3	*Mirp*	**<0.001**
Small cardioactive peptide-related peptide	*SCPRP*	**0.002**
Protein PRQFV-amide	*PRQFV*	**0.009**
Tachykinin-related peptide	*Octtkrpre*	**<0.001**
Glutathione S-transferase omega-2	*Gsto2*	0.341
Glutathione synthetase	*Gss*	0.821
Superoxide dismutase Cu-Zn	*Sod1*	**<0.001**
Peroxiredoxin 6	*Prdx6*	**0.01**
Serpin B10	*Serpinb10*	**0.001**
Glutathione peroxidase	*GPX*	**0.002**
Toll like receptor 3	*Tlr3*	**<0.001**
Fatty acid-binding protein homolog 9	Ibp-9	0.06
Rab effector Noc2	*Rph3al*	**<0.001**
Anoctamin-1	*Ano1*	0.449

*Results after MANOVA are computed after Bonferroni correction. See Figure 6 for direction of changes*.

*Bolded p values show statistically significant differences*.

## Discussion

This study provides an update of the knowledge on the complexity of the gastric ganglion of the cephalopod mollusc *O. vulgaris* using a combination of immunohistochemical and gene expression analyses. Recent transcriptome analyses of octopus and other cephalopods (e.g., Zhang et al., 2012; Albertin et al., 2015; Petrosino, 2015; Salazar et al., 2015; Liu et al., 2016; Liscovitch-Brauer et al., 2017; Tian et al., 2017) have greatly facilitated this study. The data we accessed (R. Sanges and G. Fiorito Laboratories *O. vulgaris* transcriptome; see Materials and Methods) enabled characterization of the molecular fingerprint of octopus' gastric ganglion. Despite limitations imposed by the set of transcripts considered (33 out over 60,000 in total), *in silico* gene expression analysis revealed that the ganglion is clearly distinguishable from other central and peripheral nervous system tissues, when the same set of genes is considered. This is confirmed by PCA (Figure 3), with the gastric ganglion segregated into a different quadrant in respect to the stellate ganglion, and the arm tip and the octopus' brain structures clustered in other quadrants (Figure 3).

Below we will first summarize our data and the literature to depict the neurochemical complexity of *O. vulgaris* gastric ganglion, and then discuss the effects of *Aggregata* infection on gene expression in the ganglion.

### *O. vulgaris* gastric ganglion: a contribution to the understanding of its neural complexity

In *O. vulgaris* the gastric ganglion appears as a white, oval-shaped, encapsulated structure (see Figure 1) about 3 mm long (in a 500 g body weight animal Andrews and Tansey, 1983). It is located on the serosa of the digestive tract at the junction of the crop, stomach, caecum and proximal intestine (Bogoraze and Cazal, 1946; Young, 1967). The cephalopod gastric ganglion is characterized by a dense neuropil surrounded by cell bodies, encapsulated by connective tissue. This structure was apparent from the H&E staining (Figure 4C), which also showed axon bundles exiting the ganglion for adjacent regions of the digestive tract, and confirmed previous descriptions (see Bogoraze and Cazal, 1946; Young, 1967, 1971). The dense layer of nerve cell bodies surrounds a central neuropil, with axons forming the nerve bundles linking, through an intricate network, to the adjacent structures: crop, stomach, caecum, hepatic ducts and intestine—(Bogoraze and Cazal, 1946; Young, 1967). The major nerve bundles have been identified as: superior bundle (3 nerves: anterior- and posterior-gastric, and gastro-esophageal nerves), dorsal bundle (spreading in a fan innervating the caecum), and ventral bundle (3 nerves) emerging from the posterior end (Figure 4G: *in*) of the gastric ganglion and projecting toward the intestine (see descriptions in Bogoraze and Cazal, 1946; Young, 1967).

We observed nerve cell bodies larger (external layer) than those closer (internal layer) to the neuropil (Figures 4H, 5N), in analogy to other ganglia in octopus as described by Bogoraze and Cazal (1944; 1946; see also Young, 1971). However, very small cells (less than 5 μm diameter) were not confirmed by our observations.

In the neuropil a dividing partition made of cell bodies (Figure 4J, arrowheads) was also noted, as reported by Young (1967, 1971). In *Nautilus* there are paired gastric ganglia (Owen, 1832) and we hypothesize that the partition observed in octopus' gastric ganglion is a remnant of the fusion of paired ganglia in an ancestral cephalopod to form a single ganglion in modern cephalopods.

The characterization of *O. vulgaris* gastric ganglion was further extended by identifying neuronal markers that can be utilized for studying the morphology of the cephalopod ganglia moving forward.

We identified cell bodies using NeuN antibody, a neuronal nuclear marker known to recognize neurons in the both the central and peripheral parts of the vertebrate nervous system including the autonomic innervation of the digestive tract (Mullen et al., 1992). The epitope of this antibody (see Table 1) matched at least seven nucleotide sequences (average length >1,000 bp) belonging to the *O. vulgaris* transcriptome available to us. In particular, we identified it by BLAST as the RNA binding protein fox-1 homolog 3 (RBFOX3). To the best of our knowledge, RBFOX3 gene encodes a member of the RNA-binding FOX protein family, which is involved in the regulation of alternative splicing of pre-mRNA. It has an RNA recognition motif domain, and is known to produce the neuronal nuclei (i.e., NeuN) antigen that has been widely used as a marker for post-mitotic neurons (see https://www.ncbi.nlm.nih.gov/gene/146713; see also Nikolić et al., 2013 where is reported to identify neural cells in *Helix*).

In our experiments, NeuN antibody identified a population of cells in the octopus gastric ganglion. According to the original description of Bogoraze and Cazal (1946), the nerve cells forming the cortical layers (4 to 5, *sensu* Bogoraze and Cazal, 1946) are all of “ganglionic type (plasmo- or somatochromes), large in size, abundant in cytoplasm, rich in Nissl bodies, with vesicular and nucleolus nuclei” (Bogoraze and Cazal, 1946, p. 123; Figure 4M, upper row). Following the authors' description, there are no karyo-chrome cells expected in the octopus gastric ganglion (Bogoraze and Cazal, 1946). In addition, neural cells of the cortical layers are described as placed in a neuronal “lodge” formed by fibers and agglutinated neuroglia proposed to be part of the intricate glio-vascular network characterizing typical ganglia in octopus (see Bogoraze and Cazal, 1944, 1946, p. 123). Based on the NeuN, noradrenaline and GABA immunoreactivities we observed, it is suggested that our data match with the description provided by Bogoraze and Cazal. Interestingly, the intricate network seen for noradrenaline and GABA (Figures 5I,J) resembles the original drawings of the gastric ganglion histology (see Figure XIII in Bogoraze and Cazal, 1944).

It was not possible to investigate the glia-like cells in the gastric ganglion of octopus, as specific markers are not available for cephalopods. Imperadore et al. (2017) were unsuccessful using antibodies against vimentin and glial fibrillary acidic protein in octopus neural tissues; this contrasts with previous results (Cardone and Roots, 1990). Furthermore, transcripts for glial fibrillary acidic protein in *O. bimaculoides* genome or in *O. vulgaris* transcriptome do not seem to occur (Imperadore et al., 2017).

We were unable to use synaptic markers (e.g., synapsin and synaptogamin) due to the lack of specificity of the commercial antibodies available. However, previous investigation of the *O. vulgaris* transcriptome (Zhang et al., 2012) identified the presence of synaptophysin and synaptotagmin-7 in central nervous system of *O. vulgaris*, and the genome of *O. bimaculoides* includes 13 synaptotagmin genes (Albertin et al., 2015).

*O. vulgaris* gastric ganglion cells were also positive for the vertebrate neuronal marker acetylated α-tubulin, consistent with the finding of Shigeno and Yamamoto (2002) of intense tubulinergic immunoreactivity in the gastric ganglion of the pygmy cuttlefish (*Idiosepius paradoxus*). Acetylated α-tubulin staining revealed a very intricate net of fibers that appear to contribute to the connectivity within and outside the gastric ganglion.

In *O. vulgaris* the gastric ganglion is connected to the brain by a pair of sympathetic nerves (*sensu* Young, 1967). These arise from the inferior buccal ganglion (in the supra-esophageal mass) and run to the gastric ganglion embedded in the esophagus and crop muscle, giving off branches and forming a plexus in their wall *en passant* (Young, 1967, 1971). Additionally, connection to the central nervous system is via the abdominal and intestinal nerves, which in turn connect with the visceral nerve (*sensu*, Young, 1967) originating in the palliovisceral lobe (in the posterior sub-esophageal mass). A study reconstructing the intricate intra-ganglionic network will provide a simple proof-of-concept for future studies aimed at constructing a “connectome” of the octopus brain (see, Choe et al., 2010; Marini et al., 2017).

A number of putative neurotransmitters have previously been identified in the gastric ganglion of *O. vulgaris*: dopamine (Juorio, 1971; Andrews and Tansey, 1983), octopamine (Juorio and Molinoff, 1974), noradrenaline (Andrews and Tansey, 1983), acetylcholine (Andrews and Tansey, 1983), small cardioactive peptide-related peptide (Kanda and Minakata, 2006), and octopressin (Kanda et al., 2003c; Takuwa-Kuroda et al., 2003). Receptors for cephalotocin (Kanda et al., 2003b), octopressin (Kanda et al., 2005) and gonadotrophin releasing hormone (i.e., Oct-GnRH, Kanda et al., 2006) have also been reported. To the best of our knowledge, endocrine cells have not been identified in the gastric ganglion of any cephalopod, so here we assume that the substances identified originate from neurons (or possibly supporting cells) and are neurotransmitters rather than hormones. On the other hand, Bogoraze and Cazal (1946) described a “Juxta-ganglionnaire tissue” in the gastric ganglion of *O. vulgaris* (p. 121 and following pages, Bogoraze and Cazal, 1946), and this may suggest the existence of “neurosecretory tissue” in the ganglion as shown in other neural structures of cephalopods (e.g., Barber, 1967; Bellows, 1968; Martin, 1968; Froesch, 1974).

We have extended knowledge of the diversity of signaling molecules and receptors in the gastric ganglion of *O. vulgaris*. By using *in silico* gene expression analysis, real-time polymerase chain reaction experiments and immunohistochemistry, we provide for the first-time evidence for the presence in the octopus gastric ganglion of: Cephalotocin, Corticotrophin releasing factor, FMRF-amide, Gamma amino butyric acid, Molluscan insulin-related peptide 3, Protein PRQFV-amide, Small cardiac peptide-related peptide, and Tachykinin-related peptide-like IR.

As mentioned above, the currently available commercial antibodies have been in most of the cases already used on cephalopod tissues, despite not being designed for octopus or cephalopods, and they have not been validated according to current criteria (Table 1, but see exceptions therein). The results of this study should therefore be considered as preliminary and future detailed immunohistological studies using these antibodies will require more sophisticated approaches.

We comment below briefly on each of the substances.

**Cephalotocin (CT; Figures 2, 6)**. The expression of the gene for the nonapeptide cephalotocin was demonstrated, complementing previous studies showing cephalotocin receptors (CTR_1_ and CTR_2_) in the gastric ganglion (Kanda et al., 2003b, 2005). Expression of Ov-CT was down regulated (>8-fold) in octopuses with “low” *Aggregata* parasite load (compared with those where the number of sporocysts was very limited/negligible; data not shown). Interestingly, octopus with elevated parasite load show only a limited down-regulation of CT gene expression (Figure 6), suggesting that it is further modulated in highly infected octopuses.

Cephalotocin is a member of the oxytocin/vasopressin superfamily of peptides which is widely distributed in vertebrates and invertebrates (Hoyle, 2011; Gruber, 2014), with the other member present in octopus being octopressin (Takuwa-Kuroda et al., 2003). Our findings contrast with a previous study of the gastric ganglion in *O. vulgaris* (Takuwa-Kuroda et al., 2003), which failed to show the presence of cephalotocin mRNA in the ganglion, although octopressin mRNA was clearly present. Takuwa-Kuroda et al. (2003) provided evidence for cephalotocin in the *O. vulgaris* sub-esophageal mass, which includes the lobes from which most of the extrinsic nerves supplying the digestive tract originate (Young, 1971). In cuttlefish pro-sepiatocin and sepiatocin are both found in the sub-esophageal lobes (Henry et al., 2013).

We are unable to reconcile the difference in the evidence for the presence of cephalotocin between the above octopus studies, but both provide evidence for a member of the oxytocin/vasopressin superfamily in the gastric ganglion, and the presence of the cephalotocin receptor (Kanda et al., 2003b) supports a role for a member of this family as a neurotransmitter in the ganglion. However, in *O. vulgaris*, Takuwa-Kuroda et al. (2003) found no effect of cephalotocin on rectal contractions. Cephalotocin is considered to play a role in the neurosecretory system of the vena cava (Takuwa-Kuroda et al., 2003) so it is possible that it acts as a hormone (i.e., transported via the vasculature) on the receptors identified in the gastric ganglion.

**Corticotrophin Releasing Factor (CRF; Figures 2A, 5)**. CRF-like IR has been reported in *O. vulgaris* brain tissue and co-localized with neuropeptide Y-like substance (Suzuki et al., 2003). CRF receptor has been included in the list of Class B (secretin-type) G-protein-coupled receptors in the genome of *O. bimaculoides* (see Supplementary Note 8.5 in Albertin et al., 2015). We identified a subset of neural cells belonging to the cortical layers in the gastric ganglion that were CRF-like positive, along with some positive fibers.

**FMRFamide (Figures 5M,N)**. We identified several cells and numerous fibers positive for the FMRFamide antibody utilized. There is considerable evidence for the presence of FMRFamide and/or FMRFamide-related peptides in the central (Di Cosmo and Di Cristo, 1998; Suzuki et al., 2002; Di Cristo et al., 2003; Zatylny-Gaudin et al., 2010; Cao et al., 2016) and peripheral divisions (e.g., chromatophore motorneurones, Loi et al., 1996; stellate ganglion, Burbach et al., 2014) of the cephalopod nervous system. The demonstration of pronounced FMRFamide-like IR in octopus gastric ganglion nerve fibers is consistent with other findings reporting the presence of FMRF-amide like peptides in the palliovisceral lobe (*Sepiella japonica*, Cao et al., 2016), the site of origin of the visceral nerve that connects the brain and gastric ganglion (Young, 1967, 1971), and in rectal nerve endings (*Sepia officinalis*, Zatylny-Gaudin et al., 2010) that may originate from the gastric ganglion (Young, 1967). The FMRFamide and RFamide-like peptides are widely distributed amongst the Mollusca including cephalopods (Walker et al., 2009; Zatylny-Gaudin and Favrel, 2014).

RFamides (including FMRFamide) have been implicated in inhibition of feeding in gastropods (Bechtold and Luckman, 2007) and a similar role has been proposed for cephalopods (Zatylny-Gaudin et al., 2010; Zhang and Tublitz, 2013; Cao et al., 2016), but no direct evidence has been provided. Peptide GNLRFamide increased tone, contraction frequency and amplitude in the rectum from *S. officinalis*, but was without effect on either the esophagus or contractile regions of the male or female reproductive tracts (Zatylny-Gaudin et al., 2010).

**Molluscan Insulin-Related Peptide 3 (Mirp, Figures 2A,B, 6)**. Peptides with insulin-like structures have been identified in molluscs including *Aplysia* (Floyd et al., 1999) and *S. officinalis* (Zatylny-Gaudin et al., 2016), but as far as we are aware this is the first time that a member of this family has been identified in a cephalopod ganglion. The occurrence in the gastric ganglion is consistent with a neurotransmitter, rather than the more conventional endocrine role for insulin. However, the latter has not been demonstrated in cephalopods (see Goddard, 1968).

In the mollusc *Aplysia*, food deprivation decreases insulin mRNA expression in the cerebral ganglia, and injections of insulin lowers hemolymph glucose levels (Floyd et al., 1999). However, further studies in *Aplysia* using human insulin showed that it was able to hyperpolarize neurons most likely via ion channels (Shapiro et al., 1991). Thus, a potential neuromodulatory role for molluscan insulin-related peptide 3 in the gastric ganglion should not be excluded.

It is noteworthy to report that in *Aplysia*, the same transcript has been identified as a precursor of opioid-like peptides known to be specific modulators in molluscan neurons (i.e., putative enkephalin, Moroz et al., 2006). *in silico* analysis of the *O. vulgaris* and *Aplysia californica* (Moroz et al., 2006) transcriptomes revealed that the two orthologs are very similar each other. Our gene expression experiments show that Ov-*Mirp* was downregulated in *Aggregata* infected animals (high vs. low, Figure 6). However, when gene expression data are compared against samples where the number of sporocysts was negligible Ov-*Mirp* appeared at least 8-fold upregulated in gastric ganglia belonging to octopus with “low” parasite loads, and about 3-fold upregulated in the “high” load condition (data not shown).

**Peptide PRQFVamide (PRQFV, Figures 2A,B, 6)**. This pentapeptide was first identified in *Aplysia* where immunostaining was demonstrated in axons in the wall of the digestive tract and the stomatogastric ring (Furukawa et al., 2003). In *Aplysia*, PRQFV-amide inhibits contractions of the digestive tract and modulates neurons in the buccal feeding system. The expression of Ov-PRQFV appeared significantly decreased (“high” vs. “low,” Figure 6). Octopuses with “low” *Aggregata* parasite load show an upregulation (>7-fold) of this gene when compared to animals with a limited/negligible load (data not shown). Higher levels of parasite infection induced a significant depression of gene expression (data not shown).

Based upon its location and reported functions in *Aplysia*, its presence in the gastric ganglion of *O. vulgaris* is perhaps not surprising. Incomplete PRQFV-amide prohormones have been identified in the neuropeptidome of *S. officinalis* and related mature peptides (e.g., PMEFL amide) are present in the hemolymph (Zatylny-Gaudin et al., 2016). We are not aware of functional data which would provide insights into the function of PRQFV-amide in the cephalopod gastric ganglion.

**Small Cardioactive Peptide-Related Peptide (SCPRP, Figures 2A,B, 6)**. Kanda and Minakata (2006) reported the presence of oct-SCPRP (a decapeptide) in the gastric ganglion using Southern blot, and our results provide further biological validation. A low parasite load in the digestive tract induced an upregulation of Ov-SCPRP expression, but it was reduced when the levels of *Aggregata* were elevated (comparing “high” or “low” with samples where the number of sporocysts was limited/negligible; data not shown; refer also to Figure 6).

A small cardioactive decapeptide peptide has been identified in the *S. officinalis* neuropeptidome (gastric ganglion not analyzed), but interestingly was not detected in the central nervous system (Zatylny-Gaudin et al., 2016). Contraction of the radula protractor muscle in response to SCPRP has been reported in *O. vulgaris* (Kanda and Minakata, 2006) and is consistent with a possible role for this peptide in control of digestive tract motility.

**Tachykinin-related Peptide (OcttKrpre, Figures 2A,B, 6)**. We identified various cells positive to the antibody utilized for this study (TAC1 in Table 1) distributed in the internal (smaller diameter) cortical layer of neural cells in the gastric ganglion (Figure 5O). In addition, fibers in the nerve bundles and in the neuropil, were also positive (Figure 5P). Ov-OcttKrpre appeared downregulated (“high” vs. “low,” Figure 6). However, gene expression data showed that in octopus with “low” *A. octopiana* parasite load in the digestive tract it was upregulated (when compared with samples with limited/negligible load, data not shown), confirming the view that modulation of gene expression is linked to differing parasitic loads.

Tachykinin related peptides are one of the two families of tachykinin-type peptides occurring in protostomes. Seven members of the tachykinin-related peptide family have been characterized from *O. vulgaris* (Kanda et al., 2003a, 2007) and molecular studies of the brain in both *S. officinalis* and *O. vulgaris* have identified a single precursor molecule that encodes nine peptides amidated at the C-terminal (Zatylny-Gaudin et al., 2016). The molecular data in this study extends the distribution of TKRPs to the visceral innervation in octopus. Further support for TKRPs as putative transmitters in the gastric ganglion comes from the presence of the oct-TKRP receptor (oct-TKRPR) in the ganglion (Kanda et al., 2007). The properties of the cloned oct-TKRPR have been investigated in *Xenopus* oocytes and this revealed that the receptors were most sensitive to oct-TKRP II and III. Interestingly they were insensitive to substance P (Kanda et al., 2007). Further support for TKRPs in the regulation of the digestive tract comes from stimulation of contractions in the esophagus, stomach and crop of octopus by oct-TKRP II and III (H. Minakata et al. unpublished results, cited in Kanda et al., 2007). Our results further extend IHC studies in *Nautilus* (in the heart, Springer et al., 2004) and *Sepia* (referred to as squid, Osborne et al., 1986) where Substance P-like immunoreactivity is reported for the optic lobes (processes and cells).

All the evidence for acetylcholine as a neurotransmitter in the gastric ganglion of *O. vulgaris* is indirect. It is based upon the presence of the common type of choline acetyltransferase (present study, see Figures 5G,H) and acetylcholinesterase (Andrews and Tansey, 1983). We identified a few dispersed clusters of cells in the cortical layers and several fibers in the neuropil where cChAT-IR has been identified. There is extensive evidence for acetylcholine as a neurotransmitter in other parts of both the central and peripheral components of the nervous system in cephalopods (for review see Messenger, 1996; but see also Bellanger et al., 1997, 2005; Kimura et al., 2007; D'Este et al., 2008; Casini et al., 2012; Sakaue et al., 2014) and its role as a neurotransmitter is further supported by genomic evidence for acetylcholine receptor subunits in *O. bimaculoides* (Albertin et al., 2015).

The previous studies using neurochemistry (Juorio, 1971) and histochemistry (Andrews and Tansey, 1983) to demonstrate the presence of adrenergic and dopaminergic neurons in the gastric ganglion of octopus are supported by our gene expression experiments for dopamine β hydroxylase (Dbh in Figure 6), catalyzing the reaction that produces noradrenaline from dopamine, and by the tyrosine hydroxylase IR (Figures 5A,B). In addition, Class A, rhodopsin has been reported in *O. bimaculoides* genome (Albertin et al., 2015). Acetylcholine has a stimulatory effect on the cephalopod esophagus, but an inhibitory effect (most likely via nicotinic receptors) on the crop and stomach (Wood, 1969; Andrews and Tansey, 1983) Both 5-HT and adrenaline enhance contractile activity in the crop, stomach and intestine (Wood, 1969; Andrews and Tansey, 1983).

Whilst octopamine has been demonstrated in the gastric ganglion by histochemistry (Juorio and Molinoff, 1974), it has not previously been shown using immunohistochemistry. Our study confirms its presence and distribution for the first time in the ganglion (but see for the octopus brain: Ponte, 2012; Ponte and Fiorito, 2015). Octopamine-IR is observed in bouton-like structures suggesting the existence of an intricate octopaminergic network in *O. vulgaris* gastric ganglion. This resembles findings in other invertebrates where the octopaminergic distribution in nervous structures has been described in detail (e.g., Kononenko et al., 2009). In the octopus' central nervous system octopamine-positive neurons are prominent in some lobes (i.e., basal and peduncle lobes; see Ponte, 2012; Ponte and Fiorito, 2015).

Tyrosine Hydroxylase (TH) positive neurons have been reported to be localized in discrete areas of the cephalopod nervous system including cerebral and gastric ganglia of the developing cuttlefish (Baratte and Bonnaud, 2009), and mostly in the posterior buccal lobe of the adult octopus brain (Ponte, 2012; see also Ov-Dopamine transporter in Zarrella et al., 2015), suggesting the existence of a dopaminergic modulatory system in cephalopods. In the gastric ganglion, TH-IR appears clearly in fibers and we cannot exclude the possibility that this contributes to local synthesis of final products (i.e., dopamine, noradrenaline or octopamine) as recently demonstrated in other organisms (e.g., Gervasi et al., 2016; Aschrafi et al., 2017).

GABA has not previously been reported to occur in the octopus gastric ganglion, but is widely distributed in the central nervous system (Cornwell et al., 1993; Ponte et al., 2010; Kobayashi et al., 2013). We found a distributed GABA-IR positivity revealing an intricate network appearing to surround the great majority of neural cells belonging to the external cortical layer of the ganglion. This resembled the description provided by Bogoraze and Cazal (1946), who observed “the cortical ganglion cells placed in a neuronal box formed by ‘agglutinated neuroglia’ in clear dependence with a glio-vascular network” [Our translation from French]. Noradrenaline-IR seemed to overlap partially with this GABA-IR network surrounding large neural cells. We can only speculate that this resembles the network described by Bogoraze and Cazal (1944) and that this network may represent the “juxta-ganglionic” tissue that seems to be a major component of octopus ganglia. Future studies are required to further support this hypothesis.

Recent findings extend further the role of GABA, providing evidence for functions supplementary to its classic one as a major inhibitory neurotransmitter (i.e., excitatory and inhibitory, Swensen et al., 2000; activation of glial cells, Serrano et al., 2006; “gliotransmitter,” Yoon and Lee, 2014). A close dialogue between neuro-glia modulatory systems has been also reported for norepinephrine (e.g., Gordon et al., 2005).

Functionally speaking however, neither GABA nor octopamine had an effect on the esophagus, crop or stomach in *O. vulgaris* (Andrews and Tansey, 1983).

Apart from the octopressin (OPR; Figures 2A,B, 6) and cephalotocin receptors (CTR1, Figure 2A; see also above), we identified, for the first time, cholecystokinin_A_ and cholecystokinin_B_ receptors and the orexin_2_ receptor (Hcrtr2; Figures 2A,B, 6) in the octopus gastric ganglion. CCK-like peptides and receptors are present in invertebrates (e.g., sulfakinin [SK] family, Yu and Smagghe, 2014). *In silico* studies have identified members of the CCK/SK family in molluscs (Zatylny-Gaudin and Favrel, 2014). In addition, a member of the CCK family has been identified in the brain and hemolymph of *S. officinalis* (Zatylny-Gaudin et al., 2016). The latter observation raises the possibility that the CCK receptors identified in the gastric ganglion in the present study may be responsive to CCK acting as a hormone.

The putative orexin_2_ receptor we identified (Hcrtr2, Figures 2A,B, 6) is a GPCR, 7TM domain, rhodopsin-like receptor. This finding is potentially problematic as the ligand orexin, implicated in vertebrates in food intake regulation and digestive tract motility (e.g., Kirchgessner, 2002; Volkoff, 2016), is reported to not be present in invertebrates (Scammell and Winrow, 2011). However, the orexin and allatotropin receptors are proposed to be related to each other, and allatotropins are present in protostomes (Mirabeau and Joly, 2013) including cuttlefish (Zatylny-Gaudin et al., 2016). The allatotropin receptor is not annotated in the *O. vulgaris* transcriptome as such (Baldascino and Fiorito, unpublished), but the sequence of orexin we found has a relative similarity with *Sepia*-allatotropin (55.8%; data not shown). Functional studies of the allatotropin and orexin family of peptides in cephalopods are required to characterize the putative octopus orexin_2_ receptor.

### Octopus gastric ganglion responses to *Aggregata octopiana* infection

We found changes in the relative expression of 24 genes present in the transcriptome of *O. vulgaris* when we analyzed the mRNAs of animals with relatively “low” vs. “high” *A. octopiana* loads (Figure 6). Differences were observed for genes responsible for the synthesis and release of molecules implicated in neurotransmission and in those with a potential role in inflammation and oxidative stress.

In octopus with higher levels of *Aggregata* infection, a relative increase of gene expression was found (Figure 6) for: the CCK_A_ receptor and the small cardioactive peptide-related peptide (*SCPRP*), *Nfkb2* Ov-*Tlr3. Nfkb2*is known to be the endpoint of a series of signal transduction events initiated by biological processes including inflammation, immunity, cell differentiation and growth, tumorigenesis and apoptosis. A member of the toll-like receptor family (Ov-*Tlr3*) plays a fundamental role in pathogen recognition and activation of innate immunity.

In contrast, we observed a reduced gene expression for dopamine β-hydroxylase, cephalotocin, tachykinin-related peptide, PRQFV-amide and the orexin_2_ receptor (Figure 6). This was also the case for *Litaf*, considered to play a role in endosomal protein trafficking and in targeting proteins for lysosomal degradation, thus contributing to downregulation of downstream signaling cascades.

The above functions are deduced from Universal Protein Resource (UniProt).

The paucity of functional studies in cephalopods makes it difficult to predict the functional consequences; the following discussion is speculative.

The higher expression of CCK_A_ receptors and a similar directional change in CCK_B_ receptors combined with lower levels of the orexin_2_ receptor gene is interesting since in vertebrates activation of the orexin receptor stimulates food intake (e.g., for mammals, Wong et al., 2011; for fish, Volkoff, 2016) while CCK is inhibitory (e.g., for mammals, Dockray, 2014; for fish, Volkoff, 2016). The other potential effect of the differences in gene expression would be on the movements of the digestive tract by altering gastric ganglion outputs. The reduced expression of the genes for dopamine β-hydroxylase, tachykinin-peptide related peptide and orexin_2_ receptor would be predicted to reduce the overall contractile activity of the digestive tract, which may be advantageous for the parasite to reduce expulsion. However, the relatively lower expression of PRQFV-amide is not consistent with this overall effect of *Aggregata*, assuming that PRQFV-amide is inhibitory in octopus digestive tract as is the case in *Aplysia* (Furukawa et al., 2003). Finally, SCPRP stimulates contractions of the radula in *O. vulgaris*. Our findings of increased expression levels in octopuses with high levels of infection appear to be inconsistent with inhibition of digestive tract motility.

The products of the Rab effector Noc2 gene are implicated in exocytosis and the lower levels in the gastric ganglion with increased *Aggregata* infection would be anticipated to negatively impact release of signaling molecules.

The molluscan insulin-related peptide 3 gene showed the largest difference between the two groups with relatively reduced gene expression in the highly infected group. Nothing is known of the function of this peptide in cephalopods, but a role either in regulation of food intake or regulation of metabolism appears likely.

Functional studies are required to resolve the above speculations, but the cluster of gene changes observed should focus attention on control of food intake and digestive tract motility. Whilst reduction of growth in *Aggregata*-infected *O. vulgaris* has been attributed to “malabsorption syndrome” induced by the pathological and physiological effects on the digestive tract (Gestal et al., 2002a,b), this may be further exacerbated by impaired motility and suppression of food intake resulting from effects on the innervation of the digestive tract.

Previous studies of the molecular responses to *Aggregata* infection in *O. vulgaris* have focused on the changes in the hemolymph (Castellanos-Martínez et al., 2014a,b), gills and caecum (Castellanos-Martínez et al., 2014a), but not neural tissue, although the caecum is likely to have contained some enteric neurons. In the gastric ganglion, we demonstrated a relative increase in the expression of several genes with products related to tissue inflammatory responses including *NF*κ*B2, Tlr3, Sn*, and *Serpin b10*. Toll-like receptors and NFκB pathways have previously been identified in *O. vulgaris* and the Tlr-2 appeared upregulated in hemocytes, gills and the caecum of *Aggregata* infected octopus (Castellanos-Martínez et al., 2014a). These findings are consistent with the present study and indicate a systemic inflammatory response.

Amongst the potential inflammatory mediators, the NFκB gene showed the largest magnitude relative difference (high infection > low infection) of all the genes studied with Tlr-3 receptor next. The demonstration of the expression of the Tlr-3 gene in the gastric ganglion of *O. vulgaris* is of particular relevance as the same receptor has been identified in the ENS and dorsal root ganglia of mice (Barajon et al., 2009). The relatively higher levels of Serpin B10 would be expected to reduce peptidase activity contributing to the prevention of inflammatory damage. In the hemolymph of healthy octopus, fascin mRNA (i.e., protein singed; Table 2) was expressed at higher levels than in animals with a high level of *Aggregata* (Castellanos-Martínez et al., 2014a) which is not consistent with the present study, but may indicate tissue-specific responses. It is interesting that the proteomic part of the same study showed a significant increase in fascin in the animals with a high level of *Aggregata* (Castellanos-Martínez et al., 2014b).

Among the pro-inflammatory genes, only lipopolysaccharide-induced TNFα factor was at a relatively lower level in the highly infected group. This gene has been implicated in the cephalopod immune response (Gestal and Castellanos-Martínez, 2015).

Three genes which have been implicated in oxidative stress (superoxide dismutase, peroxiredoxin 6 and glutathione peroxidase) were expressed at relatively lower levels in the gastric ganglion of animals with the higher level of *Aggregata* infection. These findings are consistent with the previous hemolymph proteomic study which showed a downregulation of peroxiredoxin in octopuses with a higher level of *Aggregata* infection (Castellanos-Martínez et al., 2014b). As reactive oxygen species are one of the host defense mechanisms, relatively lower levels in more highly infected animals may be due to the actions of the parasite to enhance its survival. *Aggregata* is an Apicomplexan parasite and this group appears to be particularly sensitive to oxidative stress (Bosch et al., 2015).

Although the difference between the groups (“high,” “low”) is clear, we do not know how long the animals in the present study had *Aggregata*, whether the changes we observed are acute or chronic, if neural tissue other than the gastric ganglion (e.g., brain) is affected by the systemic immune changes or if the changes are reversible if the infection is cleared.

## Conclusions

This combined *in silico*, molecular and immunohistochemistry study, although preliminary, has provided additional evidence for a complex neurochemical fingerprint of the octopus gastric ganglion including the identification of a number of peptide ligands/receptors for the first time in a cephalopod. By providing for the first-time evidence that the parasitic load of the digestive tract in octopus results in differences in the molecular profile in neural tissue regulating digestive tract function (i.e., the gastric ganglion), our data suggest that the possible pathophysiological effects of *Aggregata* extend beyond epithelial damage and systemic immune responses (cf. digestive parasites in mammals) to include the peripheral nervous system.

We summarized knowledge from the literature and results from the present study to illustrate the diversity of ligands and receptors in the *O. vulgaris* gastric ganglion (see Table 4) and functional effects on the motility of the digestive tract.

**Table 4:** Summary of evidence from the literature and the present study for putative neurotransmitters, neurotransmitter/hormone receptors and neurotransmitter synthesis/destruction enzymes in the gastric ganglion of *O. vulgaris*.

Ligand^*^	**References**	**Techniques**	**Receptor**	**Transmitter synthesis/enzyme release/uptake/destruction**	**Effect of ligand on *O. vulgaris* digestive tract tissue *in vitro***
**NON-PEPTIDES**					
*Acetylcholine*	Andrews and Tansey, 1983	HC, Phy		Acetylcholinesterase	Increased tone: oesophagus
	Present study	IHC		Common type choline acetyl transferase	Decreased tone/inhibition of contractions: Crop, stomach
Dopamine	Juorio, 1971	NC			
	Andrews and Tansey, 1983	HC, Phy			Tone increase: crop
	Present study	IHC		Tyrosine hydroxylase	
Gamma amino butyric acid	Andrews and Tansey, 1983	Phy			No effect on esophagus, stomach or rectum
	Present study	IHC			
5-hydroxytryptamine	Andrews and Tansey, 1983	Phy			Decreased tone and contractions: esophagus
	Present study	IHC			Increased tone contraction amplitude: intestine
Noradrenaline	Juorio, 1971	NC			
	Andrews and Tansey, 1983	HC, Phy			Contraction stimulation: oesophagus, crop, stomach, intestine, rectum
	Present study	RT-qPCR		Dopamine β-hydroxylase	
Octopamine	Juorio and Molinoff, 1974	NC			
	Andrews and Tansey, 1983	Phy			No effect on any region
	Present study	IHC			
**PEPTIDES**					
Cephalotocin	Kanda et al., 2003b	Cloning	Cephalotocin		
	Takuwa-Kuroda et al., 2003	Phy			No effect on rectum
	Present study	RT-qPCR			
*Cholecystokinin/gastrin*	Andrews and Tansey, 1983	Phy			No effect (pentagastrin) on any region
	Present study	RT-qPCR	Cholecystokinin_A_		
*Cholecystokinin*	Andrews and Tansey, 1983				No effect (pentagastrin) on any region
	Present study	RT-qPCR	Cholecystokinin_B_		
Corticotrophin releasing factor	Present study	IHC			
FMRF-amide	Present study	IHC			See discussion
*Gonadotrophin releasing hormone* (GnRH)	Kanda et al., 2006	Cloning, Phy	GnRHR		Contraction of radula retractor muscle
Molluscan insulin-related peptide 3	Present study	RT-qPCR			
Octopressin (OP)	Takuwa-Kuroda et al., 2003	Cloning, Phy			Increased tone and contraction amplitude: rectum
	Kanda et al., 2005	Cloning	OPR		
	Present study	RT-qPCR	OPR		
Orexin	Present study	RT-qPCR	Orexin receptor type 2		
PRQFV-amide	Present study	RT-qPCR			
Small cardioactive peptide-related peptide	Kanda and Minakata, 2006	Cloning			Contraction of radula protractor muscle
	Present study	RT-qPCR			
Tachykinin related peptide (TKRP)	Kanda et al., 2003a	Cloning			
	Kanda et al., 2007	Cloning	TKRPR		
	Minakata et al., unpublished (cited in Kanda et al., 2007)	Phy			TKRPII/III stimulation of contractions in esophagus, crop and stomach
	Present study	IHC, RT-qPCR			

*Non-peptides are shown in the upper part of the table and peptides in the lower half. In the absence of functional studies on the gastric ganglion data is included showing the effect of the ligand (or a closely related molecule) on tissue from the digestive tract in O. vulgaris and studied in vitro. Note that functional studies are very limited and all results, particularly negative findings, should be treated with caution until confirmed. HC, histochemistry; IHC, immunohistochemistry; NC, neurochemistry; Phy, physiological experiments; RT-qPCR, quantitative polymerase chain reaction. See section Discussion for more detailed analysis of ligands identified in O. vulgaris gastric ganglion*.

* *Italics in the ligand column indicates that evidence for the presence of the ligand is indirect based on the presence of the presumed receptor or synthetic/destructive enzymes*.

The structural and neurochemical complexity of the gastric ganglion together with its relatively large size suggest that it is not a simple relay between the brain and the digestive tract and it is likely to have an integrative role analogous to the ganglia in the stomatogastric systems of crustaceans (e.g., Swensen et al., 2000; Hedrich et al., 2011; Dickinson et al., 2015; Daur et al., 2016, and molluscs, Jing et al., 2007). Coordination of the passage of digestive tract contents from one region to another, as digestion proceeds, would be one such integrative function to explore.

## Author contributions

PA and GP conceived and designed the experiments; EB, GD, TS, and GP performed experiments; PT and CH carried out some of the experiments; GD provided *in silico* analysis of the transcriptome; EB, GD, GP collected and analyzed the data. All authors discussed the results, contributed to writing and commented on the manuscript at all stages. All authors read and approved the submitted manuscript.

### Conflict of interest statement

The authors declare that the research was conducted in the absence of any commercial or financial relationships that could be construed as a potential conflict of interest.

## Footnotes

^1^https://www.thermofisher.com/it/en/home/brands/thermo-scientific/molecular-biology/molecular-biology-learning-center/molecular-biology-resource-library/thermo-scientific-web-tools/multiple-primer-analyzer.html
